# Patient Experience and Caregiver Involvement in COVID-19 Care Pathways: Revealing System Blind Spots Through a Life-Events Calendar Approach

**DOI:** 10.3390/healthcare14121800

**Published:** 2026-06-22

**Authors:** Romain Lutaud, Juliette Mirouse, Manon Borg, Lucie Cattaneo, Jean Constance, Christian Pradier, Sebastien Cortaredona, Irit Touitou, Patrick Peretti-Watel, Philippe Brouqui, Michel Carles, Stéphanie Gentile

**Affiliations:** 1 Department of General Practice, Aix-Marseille University, 13385 Marseille, France; romain.lutaud@univ-amu.fr (R.L.); juliettemirouse1@gmail.com (J.M.); ndj.borg@gmail.com (M.B.); 2 CNRS, EFS, ADES, Aix-Marseille University, 13344 Marseille, France; 3 Service d’Evaluation Médicale, Hôpital Sainte Marguerite, Assistance Publique des Hôpitaux de Marseille, 13009 Marseille, France; lucie.cattaneo@ap-hm.fr; 4 Institut Hospitalo-Universitaire Méditerranée Infection, 13005 Marseille, France; jean.constance@free.fr (J.C.); philippe.brouqui@univ-amu.fr (P.B.); 5 Department of Public Health, Nice University Hospital Équipe d’Accueil, 06202 Nice, France; pradier.c@chu-nice.fr; 6 SSA, RITMES, Aix-Marseille University, 13005 Marseille, France; 7 IRD, SSA, MINES, Aix-Marseille University, 13005 Marseille, France; 8 Department of Infectious Diseases, Centre Hospitalier Universitaire, Université Côte d’Azur, 06202 Nice, France; touitou.i@chu-nice.fr (I.T.); carles.m@chu-nice.fr (M.C.); 9 Observatoire Régional de la Santé PACA (ORS Paca), Aix-Marseille University, 13385 Marseille, France; patrick.peretti-watel@inserm.fr; 10 Unité des Virus Émergents, UVE: Aix-Marseille University, Università di Corsica, IRD 190, Inserm 1207, IRBA, 13385 Marseille, France

**Keywords:** patient experience, care pathways, primary care, COVID-19, qualitative research, life-events calendar, diagnostic delay, mixed methods, prehospital, caregivers

## Abstract

**Highlights:**

**What are the main findings?**

- A life-events calendar biographical method identified three distinct prehospital pathway types among patients hospitalised for COVID-19, subsequently confirmed at scale in 312 patients using state sequence analysis.
- In a substantial proportion of cases, hospitalisation was triggered by a loved one rather than a clinician.

**What are the implications of the main findings?**

- A temporal dissociation between declining viral load and escalating inflammation in Stage II COVID-19 renders standard primary care monitoring tools (antigen testing, resting oximetry) structurally unable to detect the most critical phase of disease progression.
- The life-events calendar method provides a replicable mixed-methods framework for transforming patient experience data into clinically actionable pathway typologies, with relevance beyond COVID-19 to other acute conditions with insidious progression.

**Abstract:**

**Background/Objectives:** Patient experience is increasingly recognised as a key dimension of healthcare quality, yet most tools fail to capture its temporal and processual nature, limiting its contribution to system improvement. This study aimed to demonstrate how a biographical approach to patient experience can generate actionable insights for improving care pathways. Specifically, we sought to: (i) identify and characterise distinct types of prehospital care pathways among patients hospitalised for COVID-19; (ii) identify patient-perceived significant events and safety issues; and (iii) generate structured variables to inform a subsequent quantitative phase. **Methods:** We conducted semi-structured biographical interviews with 31 patients hospitalised for COVID-19 in two French university hospitals. Data were collected using a life-events calendar (LEC), enabling day-by-day reconstruction of symptoms, healthcare contacts, and decision-making processes. Thematic analysis was performed with multidisciplinary triangulation. The qualitative phase identified three pathway types and the key mechanisms underlying each; these patterns were subsequently confirmed in a separate quantitative follow-up study (*n* = 312) using state sequence analysis. **Results:** Three distinct pathway types emerged: short (≤3 days), intermediate (4–9 days), and long (≥10 days). Delayed pathways were associated with repeated false-negative tests, underestimation of severity, and silent hypoxaemia. Across all pathways, patient experience suggested critical system-level failures, including diagnostic delays and inadequate escalation of care. Notably, in many cases, hospitalisation was triggered by a relative rather than a healthcare professional. These findings highlight the role of patient and social context as key components of care pathways. **Conclusions:** When captured longitudinally, patient experience may provide actionable insights into healthcare system functioning, suggesting structural mismatches between clinical trajectories and care responses. The life-events calendar method offers a replicable framework for transforming patient experience data into clinically and organisationally relevant knowledge. Integrating such approaches into healthcare evaluation could enhance patient safety, improve care coordination, and support more responsive care systems beyond COVID-19.

## 1. Introduction

Over the past decade, capturing patient experience has become a central component of healthcare quality assessment [1]. A wide range of tools—including patient-reported experience measures (PREMs) and satisfaction questionnaires—have been developed to support this objective [2,3]. While these instruments provide valuable insights into patients’ perceptions of care, they predominantly produce aggregated and retrospective assessments, with limited ability to capture the temporal dynamics of care pathways [4,5]. They do not allow for a detailed reconstruction of the sequence, timing, and interdependencies of clinical and non-clinical events that shape health outcomes. In other words, they capture what patients experienced, but not when, in what order, or under what circumstances these experiences unfolded. This limitation is especially critical in primary care contexts, where the timing of decisions—such as seeking care, undergoing testing, or being referred to a hospital—can directly influence clinical trajectories.

Beyond their descriptive function, patient experience data hold an underexploited potential for healthcare system improvement. When collected with sufficient temporal and contextual granularity, they can help identify mismatches between patients’ trajectories and healthcare responses, detect early warning signals of deterioration, and suggest breakdowns in care coordination. From this perspective, patient experience should not be considered solely as a measure of perceived quality, but as a source of actionable knowledge for improving patient safety and redesigning care pathways. However, most existing instruments remain focused on individual perceptions at a given point in time and do not adequately account for the dynamic and relational nature of care processes. In particular, they tend to overlook the role of close social ties, despite growing evidence that relatives and caregivers (defined here broadly as partners, family members, friends, or colleagues—not necessarily pre-existing formal caregivers—who were present or involved during the acute episode) can play a critical role in recognising clinical deterioration and initiating care, especially in situations of diagnostic uncertainty. To date, no study has applied a longitudinal biographical method to reconstruct the day-by-day sequence of prehospital events leading to hospitalisation for COVID-19, nor has it examined the relational and temporal mechanisms underlying delays in care escalation.

Biographical methods, and, in particular, the life-events calendar (LEC), offer a methodological response to these limitations [6,7]. Originally developed in the social sciences to reconstruct life trajectories across multiple domains [8], the LEC was adapted in this study as a retrospective data collection tool to document the day-by-day sequence of symptoms, healthcare interactions, and patient decisions preceding hospitalisation. By structuring recall around a visual timeline, the LEC reduces memory bias and facilitates the identification of temporal proximity and causal links between events that may not emerge through conventional interviews. This process-oriented approach—focusing on sequencing, timing, and the interplay between biographical and clinical events—constitutes the core methodological contribution of this study. Unlike PREMs or satisfaction questionnaires, which capture static and aggregated perceptions, the LEC enables the reconstruction of dynamic, event-by-event trajectories—revealing not only what happened, but when, in what order, and in what relational context.

The COVID-19 pandemic provided a particularly informative context in which to apply this approach. During the first waves of the pandemic in France (2020–2021), primary care systems faced unprecedented strain. National data indicate that approximately 20% of patients with COVID-19 were hospitalised following admission through an emergency department (ED), with 11% requiring immediate intensive care [9]. Regional data from university hospitals in Marseille and Nice further showed that a majority of patients who died had initially accessed care through ED pathways [10]. These findings suggest that delays in prehospital care pathways may have contributed to adverse outcomes.

Administrative health databases, such as the French National Health Data System (SNDS), provide valuable information on healthcare utilisation but remain insufficient to reconstruct the underlying logic and temporality of individual care trajectories. They do not capture patients’ subjective experiences, decision-making processes, or the interpersonal dynamics that influence access to care. Addressing these gaps requires methodological approaches capable of integrating temporal sequencing, experiential data, and relational dimensions of care, as enabled by the life-events calendar.

This article presents the qualitative phase of a sequential mixed-methods research programme. Its objectives were to: (i) identify and characterise different types of prehospital care pathways in terms of their structure and duration, based on patients’ lived experiences; (ii) identify key events, perceived safety issues, and areas for improvement; and (iii) generate the conceptual categories required to inform a subsequent quantitative phase. This latter phase—a cross-sectional study of 312 patients using state sequence analysis—has been published separately [11] and confirmed the relevance and robustness of the pathway typologies identified in the present study.

## 2. Materials and Methods

### 2.1. Study Design

This study constitutes the qualitative phase of a sequential mixed-methods research programme [12]. In this design, the qualitative phase is conducted first, with the explicit purpose of generating the conceptual categories, variables, and measurement instruments required for the subsequent quantitative phase. Accordingly, the findings of this qualitative phase directly informed the design of the life-events calendar questionnaire used in the quantitative phase, which was deployed with 312 patients and published separately [11]. The qualitative phase was underpinned by a processual and biographical theoretical framework [6,7], which treats the care pathway as a trajectory unfolding over time and structured by sequences of interdependent clinical and social events.

### 2.2. Study Setting and Population

Patients admitted to the Infectious and Tropical Disease Departments of the University Hospitals of Marseille (IHU Méditerranée Infection) and Nice (CHU Archet) for COVID-19 between September 2020 and December 2021 were eligible. All modes of hospital entry were included (direct admission, transfer from the ED, transfer from another department, transfer from a day hospital), as were all modes of discharge. Patients, or where applicable, the families of patients, who gave their consent to participate, were interviewed. The authors had no prior personal relationship with any participant. This study is reported in accordance with the Consolidated criteria for Reporting Qualitative research (COREQ) [13] (Supplementary Table S2).

### 2.3. Sampling Strategy and Sample Size

Purposive sampling was performed by stratifying according to age group (under 50 years, 50–70 years, over 70 years) and sex, to ensure heterogeneity of prehospital pathway profiles across the sample. Recruitment continued until informational saturation was reached, defined as the point at which no new pathway types, themes, or mechanisms emerged from successive interviews [14]. Saturation was assessed jointly by the two lead researchers (a health psychologist and a general medicine physician) after each series of interviews: no new pathway types emerged after the 26th interview, and four additional interviews were conducted to confirm saturation, yielding a final sample of 31 participants. No participant withdrew after consenting. It should be noted that saturation was assessed at the level of pathway types and cross-cutting themes, not at the level of the full diversity of social situations. In particular, the long-pathway subgroup (*n* = 6) is small, and saturation for this specific configuration should be interpreted with caution; the subsequent quantitative phase [11] provided the confirmatory evidence required to establish the robustness of this typology. Within this subgroup, the structural sequence—multiple consultations without escalation, ultimately triggered by a loved one—was consistent across cases, although the specific clinical and social circumstances varied considerably between participants.

### 2.4. Data Collection

Semi-structured interviews were conducted by telephone or videoconference between August 2021 and March 2022. Interview duration ranged from approximately 45 to 90 min. Interviews were conducted by two health psychologists (Lucie Cattaneo and Jean Constance) with specific training and professional experience in biographical interviewing techniques. The same interviewers subsequently transcribed the recordings verbatim, ensuring close familiarity with the material and accurate rendering of paralinguistic elements. Interviews were audio-recorded with participants’ informed consent.

### 2.5. Interview Guide and the Life-Events Calendar Method

The interview guide (Supplementary Table S3) was developed iteratively through pretesting with five patients and refined after each pretest interview. It was structured around a biographical approach [6,7] designed to reconstruct the full sequence of clinical and social events from symptom onset to hospitalisation.

The core instrument was a life-events calendar (LEC) (Supplementary Figure S1), a retrospective data collection tool originally developed in the social sciences [8]. Adapted for clinical use, the LEC prompted patients to report, day by day from symptom onset (D0): (a) clinical events (symptoms, oxygen desaturation); (b) diagnostic pathway (testing decisions, results); (c) care pathway (GP/ED consultations, calls to emergency services, hospitalisation); (d) subjective experience (perceived severity, anxiety, sense of abandonment). This day-by-day structure served a dual purpose: it supported accurate temporal recall and revealed the precise ordering and proximity of events—which consultations preceded which tests, which symptoms preceded which clinical decisions—in a way that standard retrospective questioning cannot achieve. Following each interview, the reconstructed timeline was transferred onto the LEC grid (Supplementary Figure S1) by the interviewer, based on a re-reading of the verbatim transcript. A second researcher independently reviewed each completed grid, and any discrepancies in event positioning were resolved through discussion until consensus was reached. The interview guide also explored the patient–GP relationship, the role of loved ones, and patient-identified areas for improvement.

### 2.6. Data Analysis

Transcripts were analysed using a thematic content analysis approach following Braun and Clarke’s six-step framework [15], combining deductive coding—based on the predefined domains of the interview guide (clinical history, diagnostic pathway, care pathway, safety events)—with inductive coding to allow new themes to emerge from participants’ accounts. Coding was conducted independently by two general practitioners (Juliette Mirouse and Manon Borg) using a shared coding framework, ensuring consistency in the interpretation of clinical events. No qualitative data analysis software was used; coding was performed manually using a shared coding grid. Discrepancies were resolved through iterative discussion within the multidisciplinary research team, which integrated complementary perspectives from general practice, medical anthropology, and sociology. This process of analytical triangulation enhanced the credibility and robustness of the findings. The coding framework was refined iteratively and finalised after three rounds of revision. No formal inter-rater reliability coefficient was calculated; in keeping with qualitative research conventions, analytical rigour was ensured through intersubjective agreement, analytical triangulation, and iterative team discussion rather than statistical measures of concordance.

In parallel, a structured database was developed from participants’ narratives to extract quantifiable variables for each patient (Table 1). The translation of qualitative codes into quantitative variables followed a systematic mapping process: each thematic code was operationalised as a discrete variable (e.g., ‘loved one triggered hospitalisation’ → binary variable; ‘number of consultations’ → continuous variable), enabling structured comparison across cases. These variables were subsequently used to characterise pathway types and to inform the design of the quantitative phase questionnaire. For each participant, the reconstructed pathways were cross-validated against objective clinical data extracted from hospital records (IHU/CHU inpatient databases), ensuring consistency between reported experiences and documented clinical events.

### 2.7. Reflexivity

The study was conducted by a multidisciplinary team combining general practice, medical anthropology, sociology, and public health. Reflexivity was considered throughout the research process. The lead researcher (R.L.) is a general practitioner and was therefore aware of the potential for professional bias in the interpretation of clinical encounters—particularly regarding the evaluation of GP management—and actively sought to foreground patients’ subjective accounts rather than clinical assessments. The two interviewers (L.C. and J.C.) were health psychologists with no prior clinical relationship with participants; they were introduced to patients as researchers rather than clinicians, which helped to minimise power asymmetries inherent in patient–clinician interactions. Participants were informed that the study aimed to understand their experience and was independent of their care. Regular debriefing sessions within the multidisciplinary team were used to surface, discuss, and critically examine interpretative assumptions throughout data collection and analysis.

### 2.8. Role of This Qualitative Phase in the Mixed-Methods Programme

Beyond generating findings in its own right, this qualitative phase served three functions: (i) it identified the full range of prehospital pathway types and the key variables distinguishing them; (ii) it validated the suitability of the LEC as a data collection instrument in this clinical population; and (iii) it generated the symptom grading system (grades 1–3, based on clinical severity and management thresholds) used in the quantitative phase to construct individual symptom sequences for state sequence analysis. The quantitative phase confirmed the typology of three pathway clusters and demonstrated their association with ICU admission (adjusted OR 2.01 for the delayed cluster) [11].

### 2.9. Ethical and Regulatory Aspects

This study was approved by the French North West IV Ethics Committee (authorisation No. EudraCT/ID-RCB: 2021-A01138-33, 2 July 2021), conducted in accordance with the Declaration of Helsinki. All participants received a written information letter and a non-opposition form. Informed consent was obtained from all participants prior to the interview. Data were anonymised before analysis.

## 3. Results

### 3.1. Sociodemographic Profile of the Study Sample

Data were collected from 31 patients, of whom 16 were male. Mean age was 58.5 ± 16.4 years (range 28–87). Most participants (*n* = 21) were living with a partner and had support from at least one loved one throughout their illness. Full characteristics are presented by pathway type in Table 2.

### 3.2. Three Types of Prehospital Care Pathway

The mean prehospital pathway duration was 7.12 ± 4.3 days (range 0–16). Analysis of participants’ accounts identified three qualitatively distinct pathway types. The thresholds distinguishing them (≤3 days, 4–9 days, ≥10 days) emerged inductively from the data: they correspond to meaningful differences in events structuring each pathway, rather than being imposed a priori. These three types were subsequently confirmed and validated quantitatively in the published follow-up study with 312 patients [11].

Nearly one-third of participants (*n* = 9) were transferred to an ICU following hospital admission. Over half (*n* = 16) had COVID-19 sequelae at the time of the interview, conducted 3 to 12 months after hospitalisation.

**Short pathways (≤3 days, *n* = 9)** mainly concerned elderly and comorbid patients, closely followed by their GP. Rapid clinical deterioration was met by rapid medical response, and hospitalisation was typically triggered by the GP or emergency services.

**Intermediate pathways (4–9 days, *n* = 16)** were the most common and heterogeneous. Symptom progression was gradual, and false-negative test results frequently led to delayed diagnosis.

**Long pathways (≥10 days, *n* = 6)** predominantly concerned younger patients without comorbidities. Multiple encounters with the health system failed to produce a confirmed diagnosis; false-negative results created a persistent sense of reassurance; and loved ones were the most frequent trigger for hospitalisation. These patients were the most likely to be admitted to the ICU.

### 3.3. Cross-Cutting Themes Across All Three Pathway Types

Four themes were common to all pathway types, regardless of duration. These are presented with illustrative verbatim extracts in Table 3 and discussed below.

Theme 1: Progressive and debilitating symptom onset.

Clinical history was similar across all 31 participants. Illness onset was characterised by intense, unusual fatigue, frequently accompanied by cough or flu-like symptoms. Over the following days, the majority described progressive deterioration: asthenia became disabling, coughing worsened and interfered with eating, and the capacity to move, seek care, or make decisions became increasingly compromised. Nine participants described episodes of confusion or syncope. This progressive debilitation is important for understanding the care pathway: by the time patients recognised the seriousness of their situation, they were frequently no longer able to act independently.

Theme 2: False-negative tests as a source of mistaken reassurance.

Among the 31 participants, 9 reported at least one negative test result prior to a positive one, and 2 were never confirmed positive despite clinical presentations consistent with COVID-19. False-negative results functioned as a structural disruption in the care pathway: they led both patients and clinicians to rule out COVID-19, interrupting diagnostic vigilance at precisely the moments when it was most needed. One participant underwent four consecutive negative tests before eventually testing positive, each result reinforcing a mistaken belief that they did not have the disease. This finding was confirmed at scale in the quantitative phase, in which 10.2% of patients in the delayed cluster reported two or more negative tests before their confirmed diagnosis [11].

Theme 3: Underestimation of severity by patients and clinicians.

Most participants (*n* = 24) reported not having fully grasped the severity of their condition at some point during their prehospital pathway. This underestimation had two interacting sources. The first was the effect of extreme fatigue, which produced a state of cognitive and physical prostration in which assessing one’s own condition or initiating action became impossible. The second was iatrogenic reassurance: among the 27 patients who had consulted a doctor before hospitalisation, 7 felt that the clinician had not adequately registered the severity of their symptoms, relying on normal objective parameters (oxygen saturation, auscultation) while discounting the patient’s subjective account.

Theme 4: The decisive role of loved ones in triggering hospitalisation.

Loved ones played a decisive role in 13 of the 31 hospitalisations. This finding highlights the critical role of relatives as informal actors in the detection and escalation of care. Their intervention took two forms: direct action (taking the patient to the ED, calling emergency services) and indirect action (insisting that the patient’s GP take the situation more seriously). This pattern was most pronounced in long pathways, where patients had accumulated false reassurances and no longer perceived themselves as seriously ill. In several cases, it was the visible alarm of a partner, friend, or colleague—observing the patient from outside the progressive deterioration—that finally prompted hospitalisation. This result was confirmed in the quantitative phase, in which 37.4% of patients in the delayed cluster were hospitalised on the initiative of a family member [11].

### 3.4. Specificities of Each Pathway Type

Beyond the cross-cutting themes above, each pathway type had distinctive features in terms of patient profile, care dynamics, and the nature of events leading to hospitalisation.

Short pathways (≤3 days, *n* = 9).

Short pathways predominantly characterised elderly patients (mean age 67.3 years) with multiple comorbidities (mean 2.77), already under regular GP surveillance. In these cases, the trigger for hospitalisation was most often rapid and unambiguous clinical deterioration, and the GP played an active coordinating role. For these patients, the care pathway was short, not because of optimal healthcare system functioning, but because the severity of their baseline condition left no ambiguity about the need for urgent care.

Intermediate pathways (4–9 days, *n* = 16).

Intermediate pathways were the most common and heterogeneous. A typical pattern was one of gradual progression, with a period of apparent stability interrupted by sudden deterioration around day 7, which finally prompted hospitalisation. False-negative test results were frequent (5 patients had at least one negative test before testing positive), a pattern that was frequently associated with delayed escalation. In this group, the GP played a dual role: in some cases, effectively monitoring and escalating; in others, contributing to diagnostic delay through over-reliance on negative test results. A representative case: a man in his seventies was told by his GP that his breathing difficulties were not due to COVID-19, as a test on day 4 had been negative. That same evening, his wife took him to the ED, where he was diagnosed with COVID-19 complicated by pulmonary embolism.

Long pathways (≥10 days, *n* = 6).

Long pathways disproportionately concerned younger patients (mean age 51.2 years) without significant comorbidities. These patients were the most likely to have accumulated multiple false-negative test results, to have visited the ED before eventual hospitalisation without being admitted, and to be admitted to an ICU once hospitalised (*n* = 3). The mechanism of delay was compound: each negative test, each GP consultation returning normal parameters, and each ED visit ending in discharge reinforced both the patient’s and clinicians’ belief that the situation was not serious. Patients described reaching a state of exhausted resignation, ‘getting used to their condition’. In half of the cases, it was a loved one who finally triggered hospitalisation, often dramatically (finding the patient unconscious, observing visible cyanosis during a video call). The erratic, multi-contact nature of these pathways—many encounters with the health system, none resulting in appropriate escalation—is their defining characteristic, and the one most frequently observed among patients who subsequently required ICU admission. This was confirmed quantitatively: the equivalent cluster had twice the odds of ICU admission (adjusted OR 2.01, 95% CI 1.10–3.67) [11].

## 4. Discussion

### 4.1. Main Results

This qualitative study employed a life-events calendar approach to reconstruct the prehospital pathways of 31 patients hospitalised for COVID-19. Three pathway types emerged from participants’ accounts: short pathways (≤3 days) characterised by elderly comorbid patients and rapid GP-led escalation; intermediate pathways (4–9 days) marked by gradual progression and false-negative test results; and long pathways (≥10 days) affecting younger patients, where multiple encounters with the health system produced no appropriate escalation. A key cross-cutting finding is the role of diagnostic delay—understood here not merely as a delay in obtaining a positive test result, but as the broader gap between symptom onset and effective medical recognition of severity. This delay appears to be systemic, resulting from interactions between the patient’s own uncertainty, the limitations of available diagnostic tools, and challenges in the escalation of care within primary care settings.

### 4.2. From Qualitative Typologies to Quantitative Confirmation

A central contribution of this qualitative phase was methodological: by systematically mapping the sequence, timing, and content of clinical events through the LEC, it became possible to translate patients’ narrative experiences into structured variables. These variables formed the backbone of the subsequent quantitative phase [11], which applied state sequence analysis to 312 patients and confirmed the three-cluster typology. In particular, the quantitative phase identified a cluster of patients (29%) who sustained grade 2 symptoms for an average of 7.5 days before hospitalisation and were twice as likely to be admitted to an ICU (adjusted OR 2.01). This convergent validation across phases supports the clinical relevance of the pathway typologies identified qualitatively here.

### 4.3. The Role of Loved Ones (Informal Caregivers): An Underestimated Resource in Care Pathways

One of the most striking findings—also observed in the quantitative phase—is the important role played by loved ones in triggering hospitalisation. In 13 of the 31 cases, it was a family member or friend who ultimately triggered the emergency response. This was most pronounced in long pathways, where patients had accumulated negative signals that paradoxically reinforced their belief that they were not seriously ill. This finding suggests the need to reconsider the role of the patient in primary care: care pathways are co-constructed with a social environment, and in contexts of diagnostic uncertainty, this environment can become more sensitive to signs of deterioration than the patient themselves. Incorporating loved ones into primary care monitoring protocols—through safety-netting instructions explicitly addressed to caregivers—is a practical implication that may extend beyond COVID-19, although further research is needed to confirm this. This finding also highlights the role of caregivers as informal safety actors within care pathways, particularly in situations where clinical monitoring fails to detect deterioration. Rather than being peripheral, patient experience appears embedded within a broader relational context, in which close social ties actively contribute to the identification of warning signs and escalation of care.

Caregivers may thus function as an “informal safety net”, potentially compensating for structural blind spots in primary care, especially during phases characterised by silent hypoxaemia and misleading diagnostic signals. This perspective extends the notion of patient experience towards a more collective and relational understanding, where safety is co-produced by patients, caregivers, and healthcare professionals. This role of close social ties as informal monitors of clinical deterioration has been documented in hospital settings [16,17], but remains largely unrecognised in prehospital primary care contexts. It is important to note, however, that this informal safety function is unevenly distributed: patients who live alone, have limited social networks, or whose relatives lack health literacy may be at greater risk of delayed escalation precisely because this compensatory mechanism is unavailable to them—a dimension that future research should address explicitly.

### 4.4. A Pathophysiological Interpretation of the Three Pathway Types

The three pathway types identified in this study can be tentatively interpreted considering the established pathophysiology of SARS-CoV-2 infection. This interpretation is offered strictly as an interpretive heuristic for generating hypotheses, not as a direct demonstration from our qualitative data. We collected no biological markers from these patients—no lymphocyte counts, no C-reactive protein, no viral load. The staging model is therefore a lens, not evidence. We use it to organise the temporal patterns in the interviews. We make no claim about the underlying biology in this cohort. Siddiqi and Mehra proposed a three-stage clinical model [18] in which Stage I corresponds to early viral replication with non-specific symptoms, Stage II to localised pulmonary inflammation with lymphopenia and elevated inflammatory markers, and Stage III to systemic hyperinflammation associated with high ICU risk. Within this framework, intermediate and long pathways may reflect a critical dissociation: as viral load declines during Stage II, antigen tests may return false-negative results while inflammation continues to escalate. This mismatch between the diagnostic signal (reassuring) and the pathophysiological trajectory (worsening) may help explain why multiple negative test results and normal resting oximetry readings were associated with delayed escalation rather than prompting vigilance. The phenomenon of silent hypoxaemia [19]—in which patients maintain near-normal oxygen saturation at rest despite significant radiological involvement—is consistent with observations from our institutional cohort showing that 14.2% of patients without dyspnoea had saturation ≤ 95% [20]. These elements suggest a structural mismatch between the thresholds used in primary care to trigger escalation and the temporal dynamics of disease progression and may support the consideration of dynamic monitoring approaches—such as repeated oximetry or functional tests—for patients with persistent symptoms despite reassuring diagnostic results. This interpretation remains hypothetical. Testing it would require studies that pair day-by-day pathway reconstruction with longitudinal biological sampling.

### 4.5. Comparison with Existing Literature

Most published studies on primary care management during COVID-19 consist of clinical guideline reviews [21,22,23,24,25,26] or analyses of administrative hospital data [27,28,29,30,31], which provide valuable information on healthcare organisation and outcomes but offer limited insight into the temporal and experiential dynamics of prehospital care pathways.

Some studies have explored teleconsultation and remote monitoring strategies [32,33,34,35], primarily focusing on the effectiveness of specific interventions rather than on the processes through which patients navigate the healthcare system or the barriers they encounter along the way.

Only a limited number of studies have attempted to capture patient experience in a processual or longitudinal manner, and few have reconstructed the day-by-day sequence of events leading to hospitalisation from the patient’s perspective.

In this context, our study contributes to the literature by providing a detailed reconstruction of prehospital care pathways using a biographical approach based on the life-events calendar, combined with qualitative pathway analysis. This approach enables the identification of temporal patterns, delays, and interaction mechanisms that are often not captured in existing data sources, while preserving a high level of ecological validity.

### 4.6. Beyond COVID-19: Generalisable Lessons for Primary Care and Patient Experience Research

We studied COVID-19. But we hypothesise that some of the mechanisms we identified are not specific to it. Diagnostic delay driven by false reassurance, inadequate safety-netting, social inequalities in health literacy and healthcare access, and the under-mobilisation of primary care for patients at risk of rapid deterioration may reflect broader features of care pathways that could extend to other acute conditions characterised by insidious progression and time-sensitive escalation, although this remains to be established in each specific clinical context. Whether these mechanisms operate in other conditions is an empirical question. Our data cannot answer it. It needs direct study in each condition.

Three lessons may be transferable. First, the importance of temporality: patient experience cannot be adequately captured through cross-sectional measures alone, as the sequence and timing of events are key determinants of both care quality and clinical outcomes. Second, the role of social context: lower education levels and the absence of a healthcare professional in one’s personal network were associated with delayed hospitalisation in the quantitative phase [11], highlighting the need to address structural inequalities beyond clinical care. Third, the generative value of qualitative research within mixed-methods programmes: the qualitative phase enabled the identification of clinically meaningful categories and mechanisms that supported robust quantitative modelling. This sequential design may offer a transferable framework for patient experience research.

### 4.7. Implications for Patient Experience and Healthcare System Improvement

Our findings suggest that patient experience, when reconstructed longitudinally, may act as an early-warning signal of healthcare failure. In several cases, patients and their relatives identified signs of clinical deterioration that were not captured by standard monitoring tools or were underestimated by healthcare professionals. These findings suggest a shift from viewing patient experience as a retrospective evaluation to recognizing it as a complementary source of clinical intelligence, particularly in contexts of diagnostic uncertainty.

A key implication concerns the role of relatives, who were involved in triggering hospitalisation in 42% of cases. This finding challenges the traditional patient–clinician dyad and supports a broader conception of care pathways as socially co-constructed processes. To operationalise this, safety-netting strategies could explicitly incorporate relatives through simple and scalable interventions, such as providing written or digital alert guidance to both patients and caregivers, defining clear escalation criteria (e.g., worsening dyspnoea, persistent fever, oxygen saturation thresholds), and implementing structured follow-up contacts (e.g., scheduled telephone calls) that include the patient’s close social network when appropriate. Such approaches may enhance early detection of deterioration and reduce delays in care.

More broadly, this study suggests that qualitative patient experience data can be translated into structured and operational variables capable of informing healthcare organisation. The life-events calendar method may enable the identification of recurrent patterns of delay, false reassurance, and missed opportunities for intervention, which can guide the redesign of primary care follow-up strategies.

Finally, we point in one direction for future work. Our study was not designed to test computational methods. But patient experience data of this kind might in the future inform hybrid analytical approaches—qualitative methods, quantitative modelling, and perhaps artificial intelligence—able to detect complex temporal patterns in care trajectories. However, such approaches should be designed to complement—not replace—the interpretative and relational dimensions of care, which remain essential for understanding and acting upon patient experience.

### 4.8. Strengths and Limitations

This study has several strengths. First, it relies on an innovative biographical method specifically adapted to reconstruct care pathways, enabling a detailed analysis of the temporal sequencing of clinical and non-clinical events. Second, the sample was heterogeneous in terms of age, sex, and comorbidity profiles, allowing the identification of diverse pathway configurations. Third, patient accounts were triangulated with objective hospital records, enhancing the validity of reconstructed trajectories. Finally, the prospective validation of qualitative findings through a large-scale quantitative follow-up study [11] further supports the robustness and clinical relevance of the identified pathway typologies.

This study also has limitations. The main limitation relates to potential recall bias, as interviews were conducted between three and twelve months after hospitalisation. Although the life-events calendar structure provided a temporal framework to support recall, and previous studies suggest that salient health events such as hospitalisation are recalled with relatively high fidelity [36,37], the variability in recall delay may have affected the precision of event sequencing.

A second, and more fundamental, limitation is the exclusive inclusion of survivors. Patients who died before the interview could not participate, and their prehospital care pathways are entirely absent from this study. This represents a structural selection bias: the most severe and most rapidly deteriorating trajectories—precisely those where delayed escalation may have been most consequential—are systematically excluded. As a result, the characteristics of the long-pathway subgroup, and the mechanisms of delay described in this study, are likely underestimated in their severity and clinical impact. The pathway typologies identified here should therefore be understood as conservative descriptions of the range of prehospital experiences, and conclusions about the consequences of delayed escalation should be interpreted accordingly. Future research combining qualitative interviews with retrospective analysis of records from non-surviving patients—through proxy interviews with families or clinicians—would be necessary to address this gap.

A third limitation is the two-centre design. Although the selected centres were major referral hospitals during the pandemic, which enabled access to a wide range of clinical situations, the findings may not be fully generalisable to other healthcare settings with different organisational structures.

## 5. Conclusions

This study suggests that a biographical, temporally structured approach to patient experience—using the life-events calendar—can reveal mechanisms of delayed escalation that standard tools do not capture. Three distinct prehospital pathway types were identified, including a subgroup of younger patients experiencing prolonged, clinically insidious trajectories undetected by routine primary care monitoring. The prominent role of loved ones in triggering hospitalisation further highlights the socially co-constructed nature of care pathways, with direct implications for safety-netting practices.

These findings support a reconceptualisation of patient experience: from a static measure of satisfaction to a dynamic source of actionable knowledge for improving patient safety and care coordination. The sequential mixed-methods design—in which qualitative pathway reconstruction directly informed quantitative modelling—may offer a transferable framework for patient experience research beyond COVID-19.

## Figures and Tables

**Table 1:** Variables extracted from interviews and entered into the structured database.

Domain	Variables Extracted
Pathway duration	Number of days from symptom onset (D0) to hospitalisation
Diagnostic pathway	Number and results of COVID-19 tests; delay between first symptom and first positive test
Primary care use	Number of medical consultations; type of physician (GP/ED/other); type of consultation (in-person/telephone/video)
Clinical management	Treatments prescribed; examinations ordered; safety-netting instructions given
Symptoms	Type and onset date of each symptom; oxygen desaturation at home
Patient–GP relationship	Whether the patient had a regular GP; GP’s role in the hospitalisation decision
Social dynamics	Person who triggered hospitalisation (patient/loved one/GP/emergency service); support from loved ones
Patient experience	Perceived severity; anxiety levels (at diagnosis, before/at hospitalisation, at interview); significant events; areas for improvement

**Table 2:** Characteristics of patients and prehospital pathway duration.

	Short ≤3 Days	Intermediate 4–9 Days	Long ≥10 Days	Total
No. of patients	9	16	6	31
Average age (years)	67.3 ± 13.4	56.3 ± 15.6	51.2 ± 15.5	58.5 ± 16.1
Female sex	4	8	3	15
Living alone	2	6	2	10
Average no. comorbidities	2.77 ± 1.6	1.6 ± 1.5	1.67 ± 1.2	1.9 ± 1.6
One or more negative tests before positive	0	5	4	9
Average no. primary care contacts	1.1	1.9	2.17	1.7
GP consultation (physical)	4	11	3	18
ED consultation (discharged)	1	3	4	8
Unable to reach GP	0	2	1	3
Hospitalisation triggered by loved one	3	7	3	13
Hospitalisation triggered by GP	2	2	1	5
ICU admission	2	5	3	10
Sequelae at time of interview	4	8	4	16

**Table 3:** Cross-cutting themes in prehospital pathways: illustrative verbatim extracts.

Theme	Illustrative Verbatim Extracts
1. Progressive and debilitating symptom onset	*NK 239: “Every day I felt more fragile. I wasn’t eating anything… I started to cough, I couldn’t stand up. By Sunday, I was already knocked out, dead.”* *EP 361: “When I collapsed on the floor, I fainted with perspiration, my girlfriend picked me up.”*
2. False-negative tests as a source of mistaken reassurance	*C8: “I had done the antigenic tests, which were negative, but I thought it was strange… I said to myself, given this strange headache, I’m going to do a PCR anyway.”* *R5: “I did antigenic tests for 3 days. Every day, because I was extremely tired, so I was trying to understand.”*
3. Underestimation of severity by patients and clinicians	*C8: “I told them I wasn’t at all well, and they told me no, it’s nothing… breathing is fine, saturation is fine. They rely on their results, but not on what the patients say.”* *R5: “You don’t even think about saying I’m sick, you just think I must think about breathing, I have to think about breathing.”*
4. The decisive role of loved ones in triggering hospitalisation	*VC 112: “I was on a Zoom meeting. I was deathly pale, almost blue, and they got scared and said: ‘Call an ambulance right away’.”* *R5: “My friend broke the door. When he saw my condition, he put me in the car and went to the emergency room.”* *NK 239: “My husband called them; he said ‘my wife is going to die, she is not well at all’.”*

## Data Availability

The datasets generated and/or analyzed during the current study are not publicly available due to confidentiality and privacy considerations but are available from the corresponding author on reasonable request.

## Supplementary Materials

The following supporting information can be downloaded at: https://www.mdpi.com/article/10.3390/healthcare14121800/s1, Figure S1: Life-event calendar C-19 (day-by-day recording grid). Table S1: Specificities of the different types of pre-hospital pathways: verbatim. Table S2: COREQ checklist (Consolidated criteria for Reporting Qualitative research). Table S3: Interview guide.
