# Supplementary material for: Patient Experience and Caregiver Involvement in COVID-19 Care Pathways: Revealing System Blind Spots Through a Life-Events Calendar Approach
Source: Healthcare (Basel). 2026 Jun 22;14(12):1800. doi: 10.3390/healthcare14121800 (PMC13300091; doi:10.3390/healthcare14121800)
Supplement: Supplementary file 1 [file healthcare-14-01800-s001.zip › Figure S1.Life-event-calendar.pdf]

Supplementary Figure S1. Life-event calendar

Life-event calendar C-19

Day D0 = First sympt om	Clinical History								Health care pathway								
	Cough	Asthenia	Anosmia	Fever	Dyspnea/ Chest discomfort	Headache	Saturation test	Desaturation (saturometer)	Willingness to test	Performing a PCR test	Had the result of a PCR/Ag test (specify the result))	Self-diagnosti c questionn aire	Clinical diagnosis	GP consultatio n (specify if Family practitione r or Emergenc y Services doctor with or without a physical examinatio n)	ED consultation	Phone call to Emergen cy Health Services	Hospi talizat ion
D0																	
D1																	
D2																	
D3																	
D4																	
D5																	
D6																	
D7																	
D8																	
D9																	
