# Supplementary material for: Patient Experience and Caregiver Involvement in COVID-19 Care Pathways: Revealing System Blind Spots Through a Life-Events Calendar Approach
Source: Healthcare (Basel). 2026 Jun 22;14(12):1800. doi: 10.3390/healthcare14121800 (PMC13300091; doi:10.3390/healthcare14121800)
Supplement: Supplementary file 1 [file healthcare-14-01800-s001.zip › Table S1 Specificities of the different types of prehospital pathways verbatim.pdf]

Table S1. Specificities of the different types of pre-hospital pathways: verbatim

Themes	Verbatim
Short-duration pathways	*ADP 148: "So I got COVID, my husband got it from me, I think. When he was screened, he was fine, he had no symptoms, and suddenly a few days later, in the middle of the night I had to call the fire brigade. He had respiratory failure. So, they took him straight to the emergency room (...); they did some tests, they let him out ~~again~~. They said he was better. Two days later, in the middle of the night, it started again, so I called the fire brigade urgently. He was still suffering from respiratory insufficiency. (...) And from there they kept him in hospital for two months."*
Medium-duration pathways	*V4:" I had a headache, that's all; I did the test, so the first time it was negative ; Headaches, I couldn't feel what I was eating. I also lost my sense of smell and I lost 8 or 9 kilograms…. He examined me and told me that my saturation was low. He told me that I had to go to the hospital urgently. I went to the hospital's emergency room, and there I was found to have a pulmonary embolism, and I was hospitalized for a few days.*
Long-duration pathways	*R5 "No, actually you don't have the strength... And frankly given my state, ~~uh~~ clearly, I didn't even have the possibility to think. "*<br><br>*R5: "I feel like I've aged 10 years! All my gestures are complicated, when I say my gestures, I mean climbing stairs, walking, doing sport, everything is complicated. And then there is also weariness for many things… "*<br><br>*C8: "so they told me it was probably a flu, something, like a viral thing so uh.... They hadn't given me any antibiotics, so I went home."*
