# Supplementary material for: Patient Experience and Caregiver Involvement in COVID-19 Care Pathways: Revealing System Blind Spots Through a Life-Events Calendar Approach
Source: Healthcare (Basel). 2026 Jun 22;14(12):1800. doi: 10.3390/healthcare14121800 (PMC13300091; doi:10.3390/healthcare14121800)
Supplement: Supplementary file 1 [file healthcare-14-01800-s001.zip › Table S3- Interview guide.pdf]

Socio-demographic data

Full name or ID number

Age : |_|_|_| Sex: M □ F □ (clinical record)

Post-Covid: Start the interview by asking about their news

To date, would you say that your health has returned to what it was before you had COVID?

Quite -> Not at all

Icebreaker question

Tell me how Covid started for you? What happened from

the beginning to the hospitalization? Clinical course

What were the first signs?

When did you have a test? Tell me would you say that

your health has deteriorated?

❑ Very quickly in a few hours

❑ Quickly in less than 48 hours

❑ Gradually over several days

Once you had a positive PCR test, did anyone call you?

Were you contacted by the social security? yes/no If yes, what were the instructions and

advice apart from those concerning isolation measures?

Do you know how you got infected?

❑ Family, ❑ Professional, ❑ Health care facility EHPAD, ❑ Medical practice ❑

Not known ❑ Other specify

What type of accommodation do you live in?

- City
- Borough
- number of pieces,
- number of rooms,
- household size (family)

Management by the doctor BEFORE HOSPITALISATION

Did you have medical care before your hospitalisation? yes/no

If yes If not, why not?

❑ Via an emergency service ❑ Could not get an appointment

❑ City doctori checked, is this the

attending physician? ❑ (whether or not the

patient is known to the doctor)

❑ Didn't try to reach a doctor

❑ C15 ❑ Other specify

What role did your GP play in your hospitalisation? What is your relationship with your GP?

If contact with doctor (town or emergency service)

How many times did you visit a doctor before you were hospitalised?

Clinical examination and management

Did the doctor physically examine you yes/no

If physical examination: ❑ oxygen saturation, ❑ BP, ❑ T°, ❑ auscultation?

Did the doctor order any additional tests? yes/no

If additional examinations (specify at which consultation if more than one)

Biological check-up ❑

X-ray check-up ❑

Did the doctor prescribe a treatment which (know if the patient had a treatment other than paracetamol and which one) yes/no if yes which ones (specify at which consultation if more than one)

Did you take this treatment?

Did the doctor tell you what signs to look out for? yes/no if yes, which ones? (specify at which consultation if more than one)

Did the doctor give instructions on what to do in case of aggravation?

(specify at which consultation if more than one) (question of organising a possible follow-up)

After one of the consultations with a doctor (before being hospitalised), were you offered hospitalisation? yes/no

If yes, after which consultation if more than one? 1

er 2

ème 3

ème etc

If so, why was this not done?

Were you able to reach a doctor when you felt ill? yes/no

Did you have any contact with another health professional? If yes, which one, pharmacist, nurse, etc.?

Were you accompanied / followed by a relative? yes/no if yes, specify

Tell me about your reasons for going to the hospital/emergency department?

❑ I have been referred by my GP or city doctor

❑ I got worse without the possibility of seeing the doctor, I came by

myself.

❑ My relatives insisted that I go to hospital

❑ I got worse and called the emergency services or the fire brigade who

referred me to hospital

❑ Other specify

What was the trigger for your hospitalisation? Someone close to you? Doctor? Worrying symptom?

Mental health status

What is your level of stress or anxiety

At diagnosis 1 10

Before hospitalisation 1 10

At the time of hospitalisation 1 10

Nowadays 1 10

Significant events experienced by the patient

During your care journey, did you ever feel insecure (loss of confidence) and abandoned?

Name one positive and one negative milestone Areas for improvement

In relation to what you have experienced, what recommendations could you make?

- On care in the city
- On hospital care

What advice would you give to 'future' Covid patients?

Experience of the disease

Your experience in one sentence and then in one word
